# Structural Identification, Synthesis and Biological Activity of Two Volatile Cyclic Dipeptides in a Terrestrial Vertebrate

**DOI:** 10.1038/s41598-020-61312-8

**Published:** 2020-03-09

**Authors:** Cristina Romero-Diaz, Stephanie M. Campos, Morgan A. Herrmann, Kristen N. Lewis, David R. Williams, Helena A. Soini, Milos V. Novotny, Diana K. Hews, Emília P. Martins

**Affiliations:** 1 School of Life Sciences, Arizona State University, Tempe, AZ 85287 USA; 2 Department of Biology and Center for the Integrative Study of Animal Behavior, Indiana University, Bloomington, IN 47405 USA; 3 Center for Behavioral Neuroscience, Neuroscience Institute, Georgia State University, Atlanta, GA 30303 USA; 4 Department of Chemistry, Indiana University, Bloomington, IN 47405 USA; 5 Institute for Pheromone Research, Indiana University, Bloomington, IN 47405 USA; 6 Department of Biology, Indiana State University, Terre Haute, IN 47809 USA

**Keywords:** Chemical ecology, Behavioural ecology, Animal behaviour, Herpetology

## Abstract

Single substances within complex vertebrate chemical signals could be physiologically or behaviourally active. However, the vast diversity in chemical structure, physical properties and molecular size of semiochemicals makes identifying pheromonally active compounds no easy task. Here, we identified two volatile cyclic dipeptides, cyclo(L-Leu-L-Pro) and cyclo(L-Pro-L-Pro), from the complex mixture of a chemical signal in terrestrial vertebrates (lizard genus *Sceloporus*), synthesised one of them and investigated their biological activity in male intra-specific communication. In a series of behavioural trials, lizards performed more chemosensory behaviour (tongue flicks, lip smacks and substrate lickings) when presented with the synthesised cyclo(L-Pro-L-Pro) chemical blend, compared to the controls, the cyclo(L-Leu-L-Pro) blend, or a combined blend with both cyclic dipeptides. The results suggest a potential semiochemical role of cyclo(L-Pro-L-Pro) and a modulating effect of cyclo(L-Leu-L-Pro) that may depend on the relative concentration of both compounds in the chemical signal. In addition, our results stress how minor compounds in complex mixtures can produce a meaningful behavioural response, how small differences in structural design are crucial for biological activity, and highlight the need for more studies to determine the complete functional landscape of biologically relevant compounds.

## Introduction

Chemical signals of terrestrial vertebrates tend to be complex mixtures of compounds^1^. However, this does not necessarily mean that numerous compounds are always needed for recognition by a signal receiver (e.g.^2–4^). Single compounds, or even a selected profile from all mixture components, could be physiologically or behaviourally active in different contexts^5–8^. Intra-specific chemical signals, often liberally referred to as “pheromones” in the extensive literature, can vary considerably in their chemical structure, physical properties and molecular size^9^, and there is currently no simple way to rule out the biological roles of additional mixture components. For example, even in an extensively studied model system such as the house mouse, the biological roles of volatile ligands, compared to the lipocalin proteins that are involved in different chemosensory functions^10–13^, are relatively unknown. Using an interdisciplinary approach, here we characterise two volatile cyclic dipeptides from the complex mixture of a chemical signal in terrestrial vertebrates (lizard genus *Sceloporus*) and investigate their biological activity in intra-specific communication.

The structural diversity of compounds documented in terrestrial vertebrates is enormous^14^, and it has been difficult to associate specific structural designs or features with chemical signalling in general^1^. It has been somewhat useful to divide potential chemosignals according to their volatility: while volatile pheromones can act in longer distance signalling, protein-like molecules and other highly polar substances with very low vapour pressure (e.g. polypeptides) require direct contact between the receiver’s chemosensory structures and the signaller or their scent marks^15^. Similar considerations may apply to kairomones in predator-prey communication^5^. One common feature among some proven or putative volatile pheromonal ligands is the incorporation of nitrogen atoms into their structures^4,16–19^. However, other structurally diverse volatile chemosignals have been documented (for review, see^1,11,14^), all pertaining to terrestrial vertebrates and their thus far known semiochemistry, and there is still much to be learned about how chemical structures relate to biological function.

**Figure 1:**
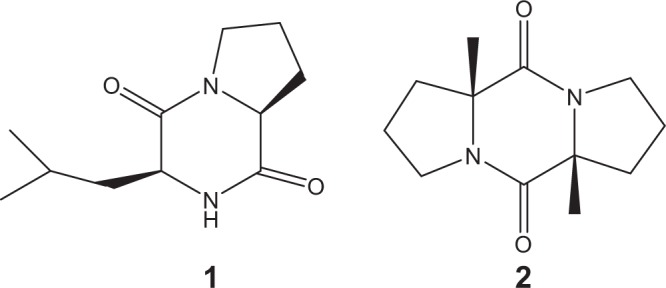
Chemical structures of commercially available cyclic dipeptide 1, cyclo(L-Leu-L-Pro) (**1**), and synthesised cyclic dipeptide 2, cyclo(L-Pro-L-Pro) (**2**).

There are two entirely different strategies to identify the physiologically and behaviourally active components of highly complex mixtures sampled from vertebrates: (i) the response-guided strategy and (ii) the chemical image strategy^20^. In the first strategy, the stimulus mixture (e.g. glandular extract) is subjected to isolation and fractionation, each followed with a bioassay, until the isolated chemical compound is structurally identified and ultimately proven as biologically active. The chemical image strategy relies on the capability to cover an entire profile of substances, assuming that many (if not all) profile constituents are involved in the complete biological response. The first strategy has particularly been fruitful in relatively simple cases such as insects^21^, while the chemical image strategy implies that enormous complexity is associated with a complete behavioural or physiological response. The downside of the response-guided strategy is that repeated fractionation of a complex stimulus-containing mixture can lead to a loss of biological activity if more than one component is needed for a robust biological response. Additionally, this approach can be procedurally tedious. From a chemist’s perspective, looking for structurally unusual compounds that consistently appear in a complex profile of substances, rather than systematically testing each and every compound, can sometimes be profitable. As we demonstrate in this study, two structurally unique compounds in a chemical mixture were positively identified from the femoral gland secretions of *Sceloporus virgatus* lizards through their mass-spectral (MS) data and a capillary gas chromatography-mass spectrometry (GC-MS) profiling technique. These mixture constituents, tentatively identified as “heterocyclic compounds” when first discovered^22^, we now report are cyclic dipeptides (Fig. 1), whose relative hydrophobicity imparts sufficient volatility to act as longer-range chemosignals.

Cyclic dipeptides, which can be classified structurally as diketopiperazines or pyrazine derivatives, have received considerable attention in recent years due to their structural stability and significant pharmacological potential related to their reported bioactivity as antibacterial, antifungal and antiviral agents^23^, but are hardly known in semiochemical roles. In nature, they are predominantly synthesised by microorganisms^24^. In animals, enzymatic pathways for production of cyclic dipeptides have been reported for the annelid worm *Platynereis dumerolii*^25^ and for the starlet sea anemone *Nematostella vectensis*^26^. Pyrazines of low molecular weights such as alkylated or alkoxylated derivatives are ubiquitous in nature. They are highly odoriferous, and not surprisingly, involved in signalling as insect alarm pheromones^27^. Another pyrazine derivative, 2,5-dimethylpyrazine, has been identified as a key component of the puberty-delaying pheromone of female mice^4,28^ and as a behaviourally relevant compound of the scent signals of male tree-shrews^29^. Moreover, different pyrazines are speculated to act as “classical alerting signals functioning as deterrents or attractants”^30^.

Among vertebrates, reptiles possess a highly developed olfactory system, characterised by the presence of the vomeronasal organ (VNO), a specialised sensory organ for processing semiochemicals^31^. The chemosensory lives of reptiles are very rich, as they use chemical cues and signals for foraging, social and spatial organization, species and sex recognition, and reproductive behaviour^32–35^, and thus chemical communication can importantly affect their fitness. One of the main sources of chemical cues in lizards are their femoral glands (FG), whose secretions are deposited on substrates as lizards move, both passively and actively^36,37^. The chemical components of femoral gland secretions (FGS), a mix of lipids and proteins, potentially serve different biological roles, not only as chemical signalling compounds, but also as structural stabilisers, antioxidants or signal enhancers^33,38–41^, yet the functions of individual compounds identified in lizard glandular secretions remain largely unknown (but see^42,43^).

Species of the large genus *Sceloporus* (90 + species^44^) are characterised by the presence of a row of femoral pores along each of their inner thighs that exude femoral gland secretions. As in many lizards^34^, both male and female *Sceloporus* use these secretions to signal individual and species identity, sex, and physiological state^36,38^, although males produce secretions more abundantly with peak production during the breeding season^36,45^. Earlier reports on FGS of *Sceloporus* list proteins, sterols and some other fairly common volatile organic compounds as part of their composition^22,39,46^. While studying evolutionary interactions between visual and chemical signals in males of four *Sceloporus* species, namely *S. cozumelae, S. parvus, S. siniferus*, and *S. merriami*^22^, we observed a number of carboxylic acids and steroids together with a series of structurally unidentified “heterocyclic compounds” with no known function. These heterocyclic compounds found in all four investigated *Sceloporus* species are the cyclic dipeptides cyclo(L-Leu-L-Pro) **1** and cyclo(L-Pro-L-Pro) **2** (Fig. 1), which can be chemically classified as diketopiperazines. We have now identified these compounds in an additional species, the lizard *S. virgatus*, and provided the synthetic analogues, one commercial and one in-house synthesized analogue, of the identified cyclic dipeptides to (i) authenticate the presumed cyclic dipeptide mixture components; and (ii) supply sufficient amounts for testing their potential biological role in intra-specific communication in a series of behavioural trials.

## Results

### Chemical composition of femoral gland secretions (FGS)

**Figure 2:**
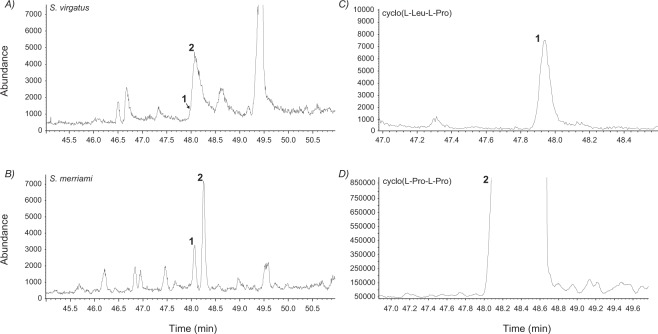
Post-run selected ion chromatogram (SIC), with m/z 70, from the lizard femoral gland extract of *S. virgatus* (**A**), *S. merriami* (**B**) and the reference standard compounds cyclic dipeptide 1 (**C**), and cyclic dipeptide 2 (**D**). Cyclic dipeptide 1, identified as cyclo(L-Leu-L-Pro), with retention time (Rt) 47.99 min and cyclic dipeptide 2, identified as cyclo(L-Pro-L-Pro), with Rt 48.12 min, are not fully resolved in *S. virgatus* but exhibit characteristic mass spectra as seen in other congeners, e.g. *S. merriami*^22^ (***B***)—shown here for comparison purposes—where they occur at higher concentrations. Peak areas for cyclic dipeptide 1 were 0.09 × 10^6^ and 0.26 × 10^6^ for *S. virgatus* and *S. merriami*, respectively. Peak areas for cyclic dipeptide 2 were 0.27 × 10^6^ and 1.1 × 10^6^ for *S. virgatus* and *S. merriami*, respectively.

**Figure 3:**
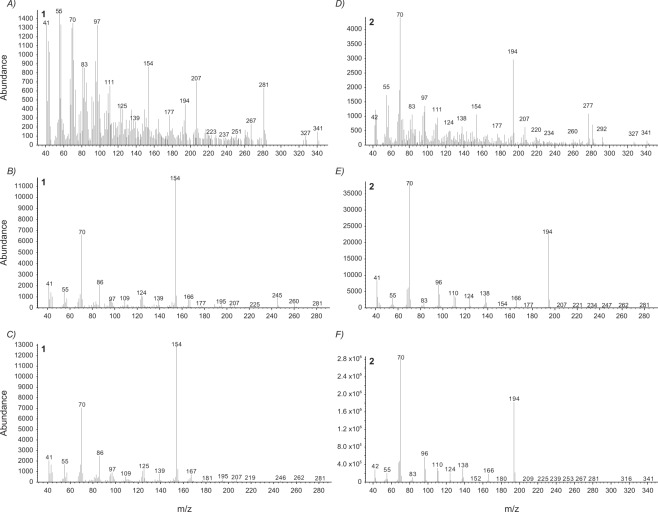
Mass spectra (electron impact, EI) for cyclic dipeptide 1 (**1**) for: *S. virgatus* (**A**), *S. merriami* (**B**), and the reference standard compound cyclo(L-Leu-Pro) (**C**). Mass spectra for cyclic dipeptide 2 (**2**) for: *S. virgatus* (**D**), *S. merriami* (**E**), and the reference standard compound cyclo(L-Pro-L-Pro) (**F**).

We identified compounds by comparing mass spectra and retention times against reference compound spectra and the National Institute of Standards and Technology (NIST) database. Samples and standard compounds were analysed by scanning the MS total ion chromatograms (TICs) in the mass range between 40–350 amu using the positive electron ionization (EI) mode as described in Pruett *et al*.^22^. After the MS recording, we extracted selective-ion currents from TICs using appropriate m/z ions as filters to obtain selected-ion chromatograms (SICs) where we measured the peak areas to compare compound abundances. The SIC peak areas were divided by the peak area of the internal standard peak area (SIC m/z 113) and by the sample weight (mg) in each sample to obtain normalised data values per weight. A total of 24 volatile compounds assigned to 8 different chemical classes were identified in the lipophilic fraction of FGS of adult male *S. virgatus* (Table 1). Short-chain fatty acids were the most abundant constituents of FGS (81.5%) and we confirmed the presence of the two volatile cyclic dipeptides in this species, cyclic dipeptide 1, cyclo(L-Leu-L-Pro), and cyclic dipeptide 2, cyclo(L-Pro-L-Pro), as shown in extracted m/z 70 ion currents (Fig. 2). Cyclic dipeptides 1 and 2 were not fully resolved in *S. virgatus* samples and we estimated peak areas using an integration approach (Fig. S1). These cyclic dipeptides were present at lower quantities than those found in congener lizard species, e.g. *S. merriami*^22^ (Figs. 2 and 3) and, overall, cyclic dipeptides were the least abundant class of compounds in FGS of *S. virgatus* (~0.1%). Generally, cyclic dipeptide 2 appeared in higher concentrations than cyclic dipeptide 1 in all FGS samples.

**Table 1:** Chemical composition of femoral gland secretions of male *S. virgatus*, in order of abundance.

Compound class	Mean %	Compounds (from more to less abundant)
Fatty acids	81.5	
saturated	51.7	Heptadecanoic acid; pentadecanoic acid; tridecanoic acid; nonanoic acid; decanoic acid; dodecanoic acid; hexadecanoic acid
unsaturated	29.7	Octadecenoic acid; 9,12-octadecadienoic acid; 9-hexadecenoic acid
Alkanes	10.1	Decane; pentadecane
Esters	3.4	Ethyl 4-ethoxybenzoate; methyl dihydrojasmonate
Salicylates	3.3	2-Ethylhexylsalicylate; homomenthylsalicylate
Alcohols	1.1	1-Hexadecanol; 2-ethylhexanol
Ketones	0.3	2-Tridecanone; 2-tetradecanone; 2-decanone
Steroids	0.2	β-Androstane
Cyclic dipeptides	0.1	Cyclic dipeptide 2, cyclo(L-Pro-L-Pro); cyclic dipeptide 1, cyclo(L-Leu-L-Pro)

### Biological activity of cyclic dipeptides

**Figure 4:**
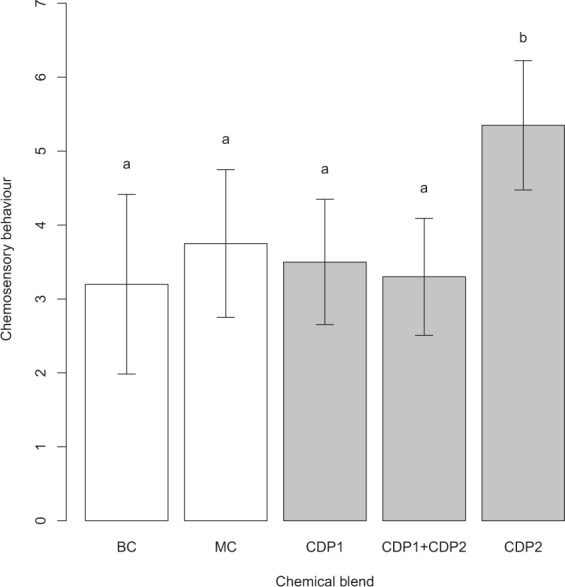
Chemosensory behaviour (number of tongue flicks, lip smacks, and substrate lickings of the pebble) of 20 male *S. virgatus* in response to a blank control (BC; an unscented pebble) and each of four different chemical blends: MC: matrix control; CDP 1: cyclic dipeptide 1, cyclo(L-Leu-L-Pro); CDP 2: synthesised cyclic dipeptide 2, cyclo(L-Pro-L-Pro); CDP1 + CDP2: a blend of CDP1 and CDP2 in equal amount. All blends included a matrix of the three most common saturated fatty acids in *Sceloporus*, in representative proportions, an acetone carrier and a non-volatile binding agent (PEG; see Methods). Shown are means ± 1 S.E. Different letters denote significantly different groups.

Chemosensory behaviour of *S. virgatus* differed among treatments during behavioural trials (*Χ*^2^_4_ = 15.08, *P* = 0.045). Lizards performed more tongue flicks, lip smacks and substrate lickings when presented with the synthesised cyclic dipeptide 2 (CDP 2) compared to the blank control (BC; coefficient estimate ± S.E.: 0.51 ± 0.16, Z = −3.28, *P* = 0.001), the matrix control (MC: 0.35 ± 0.15, Z = −2.38, *P* = 0.017), the cyclic dipeptide 1 (CDP 1: 0.42 ± 0.15, Z = −2.79, *P* = 0.005), or the combined blend of CDP1 and CDP2 (CDP1 + CDP2: 0.48 ± 0.15, Z = −3.12, *P* = 0.002) (Fig. 4). However, we found no differences between spontaneous chemosensory behaviour in the presence of an unscented pebble and the chemosensory behaviour elicited by MC (Z = 0.94, *P* = 0.347), CDP1 (Z = 0.18, *P* = 0.859) or CDP1 + CDP2 (Z = 0.52, *P* = 0.601) (Fig. 4).

## Discussion

In this study, we characterised and confirmed the presence of two cyclic dipeptides in the femoral gland secretions (FGS) of *S. virgatus*, of which at least one elicited a chemosensory response typical of social communication via olfaction and vomerolfaction^32^. Cyclic dipeptide 1, cyclo(L-Leu-L-Pro), and cyclic dipeptide 2, cyclo(L-Pro-L-Pro) are relatively hydrophobic (non-zwitterionic) dipeptides and, unlike most diketopiperazines, they are apparently detectable in the gas phase. Here, they accounted for ~0.1% of the total content of FGS. This makes *S. virgatus* the *Sceloporus* species in which these two cyclic dipeptides have been found in the lowest proportion to date^22,39^, presenting a great opportunity to test the biological activity of rare volatile constituents of complex signalling mixtures in a terrestrial vertebrate.

Even in the most studied of taxa (terrestrial mammals), it has been difficult to ascribe function to specific chemical structures^1,11,14^. For example, here, cyclic dipeptide 1 and cyclic dipeptide 2 have, relatively, very similar chemical structures (Fig. 1), including a diketopiperazine ring with nitrogen atoms, yet the biological response to each of their chemical blends was significantly different (Fig. 4); only CDP 2, when presented alone, elicited a significant chemosensory response. This disparity in the behavioural responses toward CDP1 and CDP2, together with the fact that the matrix control elicited an equivalent response to spontaneous lizard behaviour, demonstrate that the effect of CDP2 was not the result of compound class (diketopiperazine) nor compound novelty *per se*. Because the here tested compound quantities were within the naturally occurring range found in natural secretions of *Sceloporus* lizards^22,39^, it is unlikely that CDP2 acted through trigeminal chemoreception (pungency). In fact, we know that whole FGS elicit a comparable chemosensory behavioural response to CDP2, if not higher, from conspecific *S. virgatus*^39,47^, whose FGS samples contain approximately between below detection limit-282 ng of cyclic dipeptide 1 and 19–295 ng of cyclic dipeptide 2 (with m/z 70). Unexpectedly, the combined blend CDP1 + CDP2 evoked the same response as either of the controls, suggesting that CDP1 interferes with the effects of CDP2 and could mask the presence of the latter in the complete scent. However, cyclic dipeptide 2 consistently appears in higher concentrations than cyclic dipeptide 1 in the FGS of these lizards^39^ (Table 1); instead, our combined blend used an equal amount of both compounds. This allows for the possibility of CDP2 conserving its biological activity amid compounds in natural FGS. Overall, these results support the idea that biological activity resides in the nuances of structural design (i.e. it has a high specificity), relative compound proportion and/or chemical context (e.g.^48^).

To date, both cyclic dipeptide 1, cyclo(L-Leu-L-Pro), and cyclic dipeptide 2, cyclo(L-Pro-L-Pro), have been found in at least other four *Sceloporus* lizards^22,49^, the only vertebrates on the list. Cyclic dipeptide 1 has also been identified in benthic marine diatoms^50^ and different Bacteria phyla, including the mangrove rhizosphere bacterium *Bacillus amyloliquefaciens*^51,52^ and chili pepper rhizosphere bacterium *B. vallismortis*^53^ (Firmicutes), *Streptomyces spp.*^48^ (Actinobacteria), the marine bacteria *Rheinheimera japonica*^54^ and *Pseudomonas fluorescens*^55^, and *Achromobacter xylosoxidans*^56^ (Proteobacteria). Likewise, it is present in fungal cultures of *Aspergillus flavipes*^57^ and in ants^58^. Cyclic dipeptide 2 has been identified in the Antarctic psychrophilic bacterium *Pseudoalteromonas haloplanktis*^59^, the fungus *Aspergillus fungi*^60^, blowflies^61^ and bumblebees^62^. The taxonomical breadth in which these two compounds are naturally found thus seems to be quite extensive, and as diverse as the environments in which they occur. More generally, cyclic dipeptides are common by-products of anabolic and catabolic biochemical pathways, endogenous to many protists, fungi, plants and animals^63^, suggesting that these compounds may be far more frequent in animal skins^64^ and gland secretions^62,65^. A possible microbial source of cyclic dipeptides 1 and 2 within the femoral pore opening could also be considered^39,66^.

The fact that CDP 2 elicited increased chemosensory behaviour from male *S. virgatus* conspecifics suggests that cyclo(L-Pro-L-Pro) may potentially play a role in intra-specific communication in this species without the need of actual physical contact between individuals^22,47^. Furthermore, because *S. virgatus* is not the only *Sceloporus* species that excretes this compound, cyclic dipeptide 2 might also potentially operate in an inter-specific signalling context between sympatric congeners, but these hypotheses require further experimental testing. In other taxa, cyclo(L-Leu-L-Pro) has demonstrated anti-microbial and anti-mutagenic properties *in vitro*^48,52^ while cyclo(L-Pro-L-Pro) functions as a mate attractant in diatoms^50^ and has demonstrated anti-bacterial activity *in vitro*^60,61^. Thus, the fact that cyclo(L-Pro-L-Pro) could act as a pheromone in male-male communication of *S. virgatus* is congruent with previous reports of biological activity.

CDP 1 showed no apparent biological activity in intra-specific communication. There are several reasons why we may have not observed a significant behavioural response. First, behavioural responses to some pheromones sometimes require co-presentation with other constituents (e.g.^67^). Second, CDP 1 may be meaningful in other *Sceloporus* species, where increased concentrations of cyclic dipeptides in FGS occur, and its presence in *S. virgatus* is the result of phylogenetic conservatism. Third, CDP 1 may not be relevant to male conspecifics, although it may to females (e.g.^68^) or to allospecifics. Alternatively, CDP1 could modulate the effects of CDP2, as suggested by the lack of response to the combined blend CDP1 + CDP2, or it may have a structural function in FGS. For example, it may increase signal effectiveness by protecting the integrity and/or enhancing the durability of chemical scents deposited on the substrate, perhaps by slowing bacterial degradation owing to its anti-microbial effects. In ants, cyclic dipeptide 1 is putatively responsible for the bitter taste of ant venom gland secretions^58,65^. Even humans can taste relatively low levels of CDP1 (25 ppm) as metallic taste in cocoa nibs^69^. Thus, we cannot completely discard a biological role of CDP 1 and further studies are needed to discern among these and other possibilities. Follow-up studies should investigate, for example, how differences in absolute concentration, relative concentration, or the combination with additional compounds within the FGS affect behavioural responses to CDP1 and CDP2, and whether these responses differ between male and female conspecifics. To determine whether the molecular context might be important to elicit behavioural responses, it should also be instructive to present these two compounds in a different solvent, absent from FGS.

Many volatile constituents are likely by-products of general metabolism without any signalling function. In vertebrates, cyclic dipeptides (diketopyrazines) are not known in semiochemical roles and it is possible that other compounds within the FGS of *S. virgatus*, either lipids or proteins, have semiochemical activity. None of the known putative lizard pheromones, including cholesterol, cholesta-5,7-dien-3-ol and ergosterol (steroids), linoleic acid (polyunsaturated fatty acid), hexadecanol and octadecanol (alcohols), squalene (triterpene) and tocopherol (vitamin E)^33,66^ were detected in FGS of *S. virgatus* (Table 1), and thus they are unlikely to be semiochemicals in this species. In addition, we have experimentally tested other two likely candidates, namely the only steroid and the odorous ester methyl dihydrojasmonate, and found no apparent effect (C.R.D. unpub. data). In snakes, squalene and several long-chain methyl ketones (ketone) are well-characterized sex pheromones^37,66^, and the ratio of unsaturated-to-saturated ketones of pheromone blends (ranging from 10 to 18 unique methyl ketones) determines the attractiveness^70^. However, we found only two medium-chain saturated ketones in *S. virgatus*, suggesting that a similar mechanism is unlikely to operate here. Thus, any other potential semiochemicals within the FGS of *S. virgatus* remain to be identified.

In sum, we were able to characterize two cyclic dipeptides in the chemical signal of a terrestrial vertebrate, and demonstrate biological activity of cyclo(L-Pro-L-Pro), which may potentially be involved in intra-specific (male-male) communication of *S. virgatus*. This finding supports the idea that even minor components in complex mixtures can be meaningful and perhaps enough to produce a complete behavioural response^2,3,7,13^. Importantly, our results highlight the need for more detailed studies to determine the functional landscape of biologically relevant compounds in the complex mixtures of *Sceloporus* lizards, and more generally, of terrestrial vertebrates.

## Methods

### Study species

*Sceloporus virgatus* is a small (up to 70 mm, adult snout-to-vent length [SVL]) Phrynosomatid lizard that commonly occurs in Madrean pine-oak woodlands and Petran conifer forests of the Chiricahua Mountains in Arizona, USA. Like its congeners, *S. virgatus* uses multimodal communication, namely visual (motion and colour) and chemical signals in intra- and inter-specific interactions^38,47,71^. Males defend territories mainly for breeding purposes^72,73^, which they patrol, performing broadcast displays and depositing scent marks^74^, and engage in male-male competition for access to females^75^. In comparison with other *Sceloporus* species, *S. virgatus* has a higher rate of basal chemosensory behaviour and previous studies suggest that they rely more on chemical cues^47,71,76^.

### Sample collection

We collected femoral gland secretions (FGS; waxy plugs <1.0 mm diameter) from 17 adult male *S. virgatus* in the field in May 2012. We used nitrile gloves to handle lizards, and pulled waxy plugs from femoral pores on both legs using clean forceps, storing secretions in 2 mL glass vials with Teflon®-lined screw caps at −20 °C until analysis at Indiana University’s Institute for Pheromone Research. Because individual lizard samples were too small for separate chemical analyses (<1 mg^22^), we pooled secretions from various individuals to create six samples weighing 1.6 mg each and used stir bar sorptive extraction to analyse them^77^.

### Gas chromatography-mass spectrometry (GC-MS)

We characterised the volatile lipidic fraction of FGS of male *S. virgatus* using gas chromatography-mass spectrometry. The samples were weighed and placed in 20 mL glass scintillation vials, 8 ng of the internal standard 7-tridecanone (Sigma-Aldrich, Saint Louis, MO) dissolved in 5 μL methanol (Baker Analyzed®, Mallinckrodt Baker Inc., Phillipsburg, NJ), 2 mL of OmniSolv™ water (EMD Millipore Corporation, Billerica, MA) and 50 mg of ammonium sulfate (99.99%, Sigma-Aldrich, St.Louis, MO) were added to each vial. Cyclo(L-Leu-L-Pro) (99.9 + %), henceforth “cyclic dipeptide 1”, was obtained from BOC Sciences, Shirley, NY. Cyclo(L-Pro-L-Pro), henceforth “cyclic dipeptide 2”, was synthesised at Indiana University, Department of Chemistry (see details below) since pure chiral forms were not commercially available. All other reference compounds were purchased from Sigma-Aldrich (St. Louis, MO).

### Synthesis of the cyclic dipeptide 2

#### (5aS,10aS)-Octahydrodipyrrolo[1,2-a:1′,2′-d]pyrazine-5,10-dione (2)

The piperazine-2,5-dione **2** was prepared following the literature report of^78^. In our study, L-proline (23.0 g; 200 mmol) was dissolved in tetrahydrofuran (THF; 200 mL). Phosphorous trichloride (8.7 mL; 100 mmol) was dissolved in 30 mL of THF, and this solution was added into the reaction flask in approximately 10 mL quantities at 22 °C with stirring. After the addition was completed, the mixture was stirred at 22 °C for 1 h and subsequently heated to reflux for an additional 2 h. Upon cooling, the reaction mixture was concentrated under reduced pressure and water (30 mL) and then saturated aqueous sodium bicarbonate were added to adjust the pH 7–8. The precipitate was collected by filtration and washed with water (3 × 50 mL). Following silica gel column chromatography of this precipitate (methanol/ethyl acetate 1:5 by volume), the desired cyclic dipeptide **2** was obtained in 52% yield. Our bulk sample of the 2,5-diketopiperazine **2** was recrystallised three times from ethyl acetate to give fine white crystals of the pure product for biological studies.

The pure product **2** was fully characterised after drying *in vacuo*. Spectroscopic data were in agreement with the reported values^78,79^. Lit. ^1^H NMR (CDCl_3_) δ 4.16 (t, 2H), 3.49–3.54 (m, 4H), 1.88–2.33 (m, 8H)^78,79^. Mp 146–148 °C; IR (solid) 2975, 2958, 1655, 1430, 1336, 1280, 1258, 1160 cm^−1^; ^1^H NMR (CDCl_3_) δ = 4.18 (m, 2H), 3.52 (m, 4H), 2.29–2.17 (m, 4H), 2.0–1.92 (m, 4H); ^13^C NMR (CDCl_3_) δ 166.4 (C = O), 60.4 (CH), 45.1 (NCH_2_), 27.7 (CH_2_), 23.4 (CH_2_), HRMS [M + 1] calcd 195.1128; found 195.1126; [α] ^22^_D_ –145 (c 1, CH_3_OH).

### Testing of cyclic dipeptides

In May 2018, we captured 20 adult (mean SVL: 56.6 ± 0.3 mm), male *S. virgatus* by noose from a population surrounding the Southwestern Research Station (SWRS) in Cochise County (AZ, USA). We housed them individually in glass terraria (50.8 × 27.9 × 33.0 cm) containing a paper substrate and a wooden perch in the Live Animal Holding Facilities at SWRS. Terraria were placed on shelves in a screened concrete porch and hence received indirect sunlight and were subjected to natural daily variation in ambient air temperatures. Terraria were misted with water every two days, and a 60 W lamp located towards one end of the terrarium provided heat on a 12:12 h light:dark photoperiod. Lizards were visually isolated from one another, fed two crickets every other day and allowed 48 hours of acclimation to captivity before the beginning of behavioural trials, which occurred in their home terraria.

We presented each lizard with four different chemical blends and a blank control, in random order. One of the chemical blends, the matrix control, was composed of 2 mL of acetone, a fatty acid matrix with the three most common saturated fatty acids found across *Sceloporus* secretions in representative relative proportions^22^ (i.e. 25 µL tetradecanoic acid, 150 µL hexadecanoic acid and 50 µL octadecanoic acid, corresponding to 250 ng, 1500 ng, and 500 ng in the applied 20 μL of test solution, respectively), and 60 mg of polyethylene glycol (PEG). The other three treatment blends, additionally included 50 µL of one or each of the two cyclic dipeptides of interest diluted in acetone, with each corresponding to 500 ng in the applied 20 μL of test solution. These tested compound quantities are within the naturally occurring range found in FGS samples of *Sceloporus* lizards (i.e. cyclic dipeptide 1: 12–529 ng; cyclic dipeptide 2: 19–791 ng)^22,39^. The saturated fatty acids on the blend’s matrix are also very common in FGS of other lizard taxa and are associated with a structural, non-informative function^33,80^. PEG is a non-volatile, odourless, and colourless polymeric binding agent that entraps temporarily the volatile compounds in the blend. Due to their hydrophobic and volatile nature, cyclic dipeptides were not presented alone. By embedding the cyclic dipeptides in the matrix control we were able to test the effects of these compounds in analogous conditions to those in which they appear in nature while avoiding their premature evaporation during transfer onto the cue surface.

Thus, to one of the treatment blends, hereafter “CDP 1”, we added the commercially available cyclic dipeptide 1, cyclo(L-Leu-L-Pro); to a second blend, hereafter known as “CDP 2”, we added the laboratory synthesised cyclic dipeptide 2, cyclo(L-Pro-L-Pro) (see above). We made a third blend by adding an equal quantity of each of the two cyclic dipeptides (“CDP1 + CDP2”). The fourth blend acted as a matrix control (“MC”) and had no added cyclic dipeptides, but contained acetone, the fatty acid matrix and PEG (see above). Blends were mixed in capped glass vials, stirred homogeneously using a vortex and stored at 4–6 °C until use. Wearing nitrile gloves, we used a 50 µL Hamilton syringe (Hamilton Company, Reno, NE) to apply 20 µL of treatment solution onto a pebble and deposited it inside the lizard’s terrarium on top of a 15 × 15 cm glazed tile. We cleaned the syringe and pebbles with acetone between applications. In the blank control treatment (hereafter “BC”), we replicated this presentation procedure but deposited an unscented pebble with no added test solution. Upon presentation, we video-recorded lizard behaviour during 15 min and later scored chemosensory behaviour, namely, the number of tongue flicks, lip smacks, and substrate licking (directed at the pebble; Table S1). Chemosensory behavioural acts involve gustation, olfaction, and vomerolfaction in lizards and their frequency reflects the strength of the response to a particular chemical stimulus^34,81^.

All procedures described adhere to national and international guidelines for the ethical use of animals in research and were approved by Arizona State University Institutional Animal Care and Use Committee (protocol 17-1597 R to E.P.M.). Animal collection was permitted by Arizona Game and Fish Department (LIC #SP621793) and the US Forest Service.

### Statistical analyses

To test for differences in the response to different chemical blends, we analysed the scored chemosensory behaviour in R statistical software^82^, using generalised linear mixed models (GLMM). To account for repeated measures of the same individual we used individual ID as a random factor. We used package lme4^83^ and models with a Poisson distribution and a log link. We used pairwise post-hoc comparisons with a Holm-Bonferroni correction^84^ and verified model assumptions on the residuals.

## Supplementary information

Supplementary Information.
