# Supplementary material for: Structural Identification, Synthesis and Biological Activity of Two Volatile Cyclic Dipeptides in a Terrestrial Vertebrate
Source: Sci Rep. 2020 Mar 9;10:4303. doi: 10.1038/s41598-020-61312-8 (PMC7062908; doi:10.1038/s41598-020-61312-8)

**Supplementary Information**

Structural Identification, Synthesis and Biological Activity of Two Volatile Cyclic Dipeptides in a Terrestrial Vertebrate

Cristina Romero-Diaz^1,*^, Stephanie M. Campos^2,3^, Morgan A. Herrmann^1^, Kristen N. Lewis^4^, David R. Williams^4^, Helena A. Soini^4,5^, Milos V. Novotny^4,5^, Diana K. Hews^6^ & Emília P. Martins^1^

^1^School of Life Sciences, Arizona State University, Tempe AZ 85287, USA

^2^Department of Biology and Center for the Integrative Study of Animal Behavior, Indiana

University, Bloomington IN 47405, USA

^3^Center for Behavioral Neuroscience, Neuroscience Institute, Georgia State University, Atlanta GA 30303, USA

^4^Department of Chemistry, Indiana University Bloomington IN 47405, USA

^5^Institute for Pheromone Research, Indiana University Bloomington IN 47405, USA

^6^Department of Biology, Indiana State University, Terre Haute IN 47809, USA

^*^Correspondence: [cromerod@asu.edu](mailto:cromerod@asu.edu)

**Table S1.** Ethogram of chemosensory behaviour.

| Behaviour | Description |
| --- | --- |
| Tongue-flick | Each time the tongue protrudes from the lizards’ mouth, then goes directly back into the mouth. |
| Lip smack | Each time the lizard opens and subsequently closes its mouth. |
| Substrate lick | Each time the tongue protrudes from the lizards’ mouth, contacts the substrate, and then goes directly back into the mouth. |

**Figure S1A.** Total ion chromatogram (TIC) of the femoral gland secretion of *S. virgatus*.

Arrows indicate the cyclic dipeptides 1, cyclo(L-Leu-L-Pro), and 2, cyclo(L-Pro-L-Pro), at low abundance.

Abundance

**1**

**2**

**Time (min)**

**Abundance**

**Figure S1B.** Post-run selected ion chromatogram (SIC) m/z 70 from the Fig. S2A TIC graph.

Cyclic dipeptides 1 (Rt 47.99 min) and 2 (Rt 48.12 min) are not fully resolved, but exhibit characteristic mass spectra.

**1**

**2**

**Time (min)**

**Abundance**

**Figure S1C.** Integration approach for the unresolved peaks of cyclic dipeptides 1 and 2.


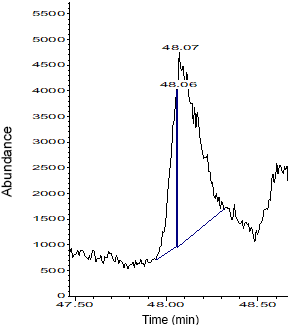

Supplement: Supplementary file 1 — Supplementary Information. [file 41598_2020_61312_MOESM1_ESM.docx]
